# Expanding emotional capital in court

**DOI:** 10.3389/fsoc.2022.1078813

**Published:** 2022-12-23

**Authors:** Cecilia Y. Nordquist, Stina Bergman Blix

**Affiliations:** Department of Sociology, Uppsala University, Uppsala, Sweden

**Keywords:** criminal court, elite professions, emotions, emotional capital, interaction, judges, prosecutors, emotion management

## Abstract

This article develops the concept of emotional capital by exposing its operation in proceedings between legal elite professionals. We argue that (a) the micro-structural restraints of the interaction order among the participants have to be accounted for in order to understand the dynamics of emotional capital, and; (b) the emotional processes at play have to be expanded beyond feelings of care showing how emotions can be employed to reproduce status and power. Empirical examples from criminal courts in Scotland and the United States demonstrate that judges and prosecutors depend on emotional capital to steer the legal proceedings. Emotional capital is both stable in that acquired capital often can be transferred across fields and volatile in that it presupposes interactional agreement to ensure successful emotional capital employment. In contrast, the lack of such agreement may devalue emotional capital regardless of overall capital wealth. In high status bureaucratic positions, the conversion of emotional capital into symbolic capital not only affects the authority of individual actors but reproduces public trust in governmental institutions.

## Introduction

“[chuckles] Yes, I'm gonna be honest with you. You can see from my body language if I'm gonna be polite with you. If you're kind or you're snooty. [If you're snooty] I stand back; I'll be short, not talking to the person. If you're kind, I'll be like LET'S TALK. Because I usually… I think their purpose of coming to me as some form of an enemy, or to beat me as opposed to work to resolve an issue is just [bullshit]” (Prosecutor Lowell, 50+ years old, African American, field notes, First Instance Court, United States).

Above, Lowell, a prosecutor with long court experience, reflects on how he manages different defense lawyers on a daily basis by adapting his emotional display to that of the approaching lawyer. As a prosecutor, he is dependent on the collaboration of other actors to gain information (“work to resolve an issue”), but he also demonstrates his power position through his strategic use of emotional display. Emotion becomes a resource, a form of capital. Lowell refers to feelings usually associated with emotional capital, such as being kind, but his more powerful display is of unwillingness to interact (“[If] you're snooty. I stand back, I'll be short”), involving emotions of irritation and reluctance. Lowell's reflections on strategically using emotional displays as professional tools indicate emotional engagement toward resolution rather than conflict (“to beat me”). An engagement backed up by organizational calls for efficient case management.

This paper will explore the concept of emotional capital by putting it to use in a professional and bureaucratic elite setting arguing that we need to (a) account for the micro-structural restraints of the interaction order to understand the dynamics of emotional capital, and; (b) widen the emotional processes at play beyond feelings of care and empathy since emotions can demonstrate status and power as well. The latter aligns with the Bourdieuian theory of capital, since it elucidates how emotional capital can convert to and from other capital forms. Elite professionals are often rich in capital, bringing the link between macro-structural positions and micro-resources to the fore (c.f. Hochschild, 1979). However, as we will argue, the example from elite professions also highlights the volatility of emotional capital in everyday interactions.

The discussion of emotional capital reappears tidally in the fields of sociology and education. In the early 1980s, emotional capital, an extension of Bourdieu's capital theory, was considered an inherently feminine capacity, employed when lacking other capital forms. In these early publications, emotional capital was conceptualized as growing out of social capital, tightly linked to relations in private life or in care and educational occupations (see Nowotny, 1981; Allatt, 1993; Reay, 2000; Zembylas, 2007). In recent years, emotional capital has instead been associated with cultural capital, a change that turns the focus to behavioral skills and thereby has widened the range of occupations where this capital form is valued, e.g., politics and research, including men to a larger extent (Cottingham, 2016). The transformation of the definition and role of emotional capital can both be linked to a growing interest from researchers in exploring emotions and to an emotionalization of society (Karstedt, 2002; Holmes, 2010), in which emotion has become a valued resource (Illouz, 2007) and a key tool used to exercise power (Heaney, 2019).

Definitions of emotional capital have varied in both what this capital form includes and how actors can produce and convert it to other forms. In an overview of the evolution of the concept Marci D. Cottingham defines emotional capital as “a tripartite concept composed of emotion-based knowledge, management skills, and capacities to feel that links self-processes and resources to group membership and social location” (Cottingham, 2016, p. 452). Departing from Cottingham's definition, we widen the emotional repertoire at play whilst accounting for the role of social interaction to promote a more nuanced theorizing of emotional capital. We argue that the ephemeral nature of emotions ties emotional capital to the interaction order (Collins, 2004), and that this volatility needs to be incorporated in a conceptual understanding of how emotions can be used for capitalization.

In other words, where Cottingham focuses on the tension between agency, structure, and gender in her elaboration and expansion of emotional capital, we aim to highlight the interactional agreement needed to ensure a successful employment of capital and how the lack of such an agreement can devalue capital regardless of overall capital wealth. What are the interactional dynamics of emotional capital, and which emotions can be utilized as capital in high status professions?

Since previous research on emotional capital with a few exceptions (Cahill, 1999; Heaney, 2019), has focused on traditionally feminine practices, the court is a suitable arena to expand the investigations of emotional capital for several reasons. First, the court engages high status actors with a less prevalent gender divide. Second, professional practice in court involves a wide array of emotions that have previously not been associated with emotional capital, in particular emotions such as ease and irritation. Third, although the legal arena builds on rules and regulations, legal practice relies on collaboration between professionals and with lay participants (Bergman Blix and Wettergren, 2018a). Furthermore, the legal professions serve as the epitome of a bureaucratic professional ideal, making studies into their employment of emotional capital an interesting case in point for understanding elite professions in general. In the following, we outline our theoretical framework integrating capital theory with social interactionist perspectives, and emotion sociology. Next, we present the methods and material, followed by our analysis of observations of and interviews with judges and prosecutors and a conclusion.

### Emotional capital in social interaction

A foundation of Bourdieu's (1990) theoretical framework concerns how people's social position is influenced by both objective structures and structural constraints. Objective structures refer to practices, such as language and economy, which we all have to relate to and abide by regardless of our position (Bourdieu, 2018, p. 3), while structural constraints, such as gender and class, constitute the foundational social positions we hold in society. In this article, we zoom in on groups holding a privileged social position in society, judges and prosecutors. Their social position, or habitus, implies that they are able to employ different forms of capital in order to navigate interaction. Habitus, that is our embodied internalization of society, constitutes the basis of capital theory, and links social position to interaction, since it provides actors with a “feel for the game” (Bourdieu, 1990, p. 63).

#### Habitus and strategy in interaction

This top-down perspective, where positions shape interaction can seem contradictory to an interactionist perspective that puts the situation at the heart of social order (Blumer, 1969). However, as argued by Bottero and Crossley, the importance of interaction can also be seen as implicit in Bourdieu's model, as “the mechanism by which social position shapes habitus” (Bottero and Crossley, 2011, p. 102). How would people acquire their habitus specific taste or learn how to act in accordance with, “habituate” (see Section Emotion at work) their social position if not in repeated interaction with others with similar capital profiles? Socialization as a base for shaping habitus bridges the analytical divide between “relations” as referring to matches or mismatches between people's social position (Bourdieu, 2018), vs. “relations” as referring to qualitative ties between people (Bottero and Crossley, 2011).

If socialization is a means to form habitus and accumulate capital, this embodied “feel for the game,” also needs to be negotiated and performed in face-to-face interaction, by fitting “one's own act to the on-going activities of others” (Blumer, 1969, p. 97). Bourdieu conceptualizes people's momentary ability to interpret and navigate a social space as “strategy”; understood as a kind of practical sense habituated by the actor. “[E]ven the most strictly ritualized exchanges […] have room for strategies” (Bourdieu, 2018, p. 15); or from an interactional perspective, joint action demands all social actors to interpret and evaluate situations in order to reach one's goals and remain a steady player in the game. This is because participants depend on a shared working consensus on what the situation is about for their actions to make sense (Goffman, 1983) in effect depending on an “interaction order sui generis” (Rawls, 1987). This need to trust others makes interactions inherently vulnerable; participants' continuous need to tune into and adapt to the evolving situation may fail or mismatch (Goffman, 1983, p. 4):

… to say that we are thus made vulnerable is also to say that we command the resources to make others similarly vulnerable to us; and neither argument is meant to deny that there might not be some conventional specialization, especially along gender lines, of threatened and threatener.

As depicted by Goffman, relational power structures put some people's definitions of the situation above others providing certain actors more room to maneuver but all actors need to protect their exposed social selves (Rawls, 1987). Furthermore, in institutional settings such as the court, interactions are often “strictly ritualized” (Bourdieu, 2018, p. 15). Judges' professional position in the center of these rituals, presiding over when and how participants shall partake, can alleviate their situational status, owing to accumulated high levels of emotional energy (Collins, 2004). Habitual expectations and institutional regulations stabilize interactions through accumulated (high or low) levels of emotional energy. As we will argue, court hearings still constitute an institutionalized volatile interaction order due to different expectations of lay and professional actors. Legal professionals hold procedural knowledge and a central position in the interaction, while lay people often lack said knowledge but have high personal stakes involved. Before we develop on the concept of emotional capital in more detail, we need to elaborate on a sociological perspective of emotions at work.

### Emotion at work

Although much debated, Hochschild's (1983) notion of emotional labor still stands strong in contemporary sociology. She turned emotions, previously associated merely with psychological processes, happening inside people, into social phenomena, happening between people. This move allowed for analyses of the structural patterning of emotions linked to different expectation and norms for different groups of people, thus requiring work to fit with societal expectations. Emotions are not just something people have; it is also something people do.

People work on or manage their emotions contingent on the norms or “feeling rules” (Hochschild, 1983) of situations and relationships. A judge feels and acts in different ways depending on if she is at home with her family or in court chairing a hearing. Situations and relations can also pose different requirements on coherence between emotional experience and display. In some situations, a display without any grounding in an experience “surface acting” can be sufficient, while other situations demand “deep acting” (Hochschild, 1983), where emotional experience is congruent with the display. Professional work, closely linked to identity, generally have high expectations on “deep acting” (Fournier, 1999).

Research into the management of emotions at work has primarily focused emotions with articulate and often deliberate display, such as smiling to customers (Gerrard, 2019), caring for patients (Diefendorff et al., 2011), or using anger to promote subordination (Bhowmick and Mulla, 2016). Especially in relation to care work, feeling with customers, patients or clients—being empathic—has also been an important dimension of understanding emotional labor. Empathy is “a process of emotionally tuning into others' emotions while imaginatively taking the perspective of the other” (Bergman Blix, 2019, p. 164), inherently linking the employment of empathy to an ongoing interaction.

Lately, sociological research has started to explore more low-key emotional processes, such as interest, irritation or doubt that usually work in the background without clear physical correlates (Barbalet, 2011). These emotions, intertwined with cognitive processes, are often linked to professional work; a judge needs to feel ease in order to lead a proceeding and prosecutors need to feel certain about their decision to indict. For novice professionals, these emotions can demand hard work to acquire but can settle into habituated patterns with practice and experience. Habituation, defined as the performance of emotion “without conscious manipulation” (Bergman Blix, 2015, p. 3), is part of the socialization of emotion and considered a prerequisite to align with and uphold a professional script. Habituation aligns individual experience and social expectations; backgrounding emotion and emotion management (Barbalet, 1998). In other words, habituation of emotions in accordance with the feeling rules of a particular field can build cultural capital. This is because managing and displaying emotions in accordance with, in our case, a professional script awards status and builds confidence to increase the likelihood of reproducing status (Kemper, 2006, p. 101–2):

A history of more or less successful interactions (i.e., where one has received status as desired and has had adequate power) leads to a general expectation of good outcomes, or optimism. Frequent failures in these areas lead to a general expectation of poor outcomes, or pessimism. Confidence depends on an appraisal of one's resources in relation to the future interaction at issue. If the setting, the interaction partner, and other features augur success, then confidence ensues, otherwise, there will be lack of confidence.

As described by Kemper, emotions are conditioned by the interaction in which they unfold, making their suitability dependent on the momentary interplay with other actors (Goffman, 1967). Also high status actors can lose confidence and risk their status by repeated failing interactional experiences. With these emotion theoretical tools in mind, we will elaborate on the linkage between emotional capital and social interaction.

#### Capital and emotional practices

The foundation of capital theory is that all capital forms, economic, cultural, social and emotional, are resources to acquire prestige, or *symbolic capital*, defined as recognized competence (Bourdieu, 1986a). This means that for economic wealth or social relations to become capital, they need to be recognized as valuable and possible to convert. By accumulating and converting capital, actors obtain and cement different structural positions. Bourdieu differentiates between two types of conversions: the instantaneous, rooted in financial logic, and the social, requiring time and social relations to turn into capital (Bourdieu, 1986a, p. 24). For example, a junior judge might have to work harder to acquire prestige in the courtroom than a senior judge. Although they hold the same role professionally, the senior judge has converted time on the bench into symbolic capital. When conversion processes take time, their value becomes more uncertain, requiring strategies to a greater degree (Bourdieu, 1986a). One could object that since judges belong to the prestigious and powerful legal field, their structural position would cement their symbolic capital without dependence on interactional strategy. Although a judge in court is vested with structural power by her professional position, she also needs situational status and power to lead the proceedings and collect evidence (Bergman Blix and Wettergren, 2018a). This is because the stress on performance highlights the fact that legal professionals need to *embody* the symbolic power or prestige necessary to lead the legal process forward, they need to display their status (see further Persson, 2021). As depicted in the introductory quote, they need to convey certain emotions, such as irritation at ill-prepared lawyers, or ease toward nervous witnesses, while hiding or toning down others. Social position matters here, an older male judge may be granted more status independent of performance, but previous studies show that also they depend on their ability to adapt their emotional display to manage upcoming situations (Bergman Blix and Wettergren, 2018a).

Furthermore, as depicted above interaction depends on a basic agreement on how to interpret what the situation is about and what is at stake (Goffman, 1986). In a court setting, the lay people coming to court often do not understand or disagree with the legal understanding of the situation leading them to act in inappropriate ways or refuse to cooperate. This can jeopardize the moving forward of the process as well as the collection of material for legal interpretation and judgment. The different legal professionals share a basic agreement on “what is going on here” (Goffman, 1986, p. 8), but they have reason to disagree on interpretation for strategic reasons (Bourdieu, 1986a). Both demand emotional attuning and empathy to forestall and counteract controversy and to collect valid material for decision-making. The situational and adaptive character of emotions (see further Scheer, 2012, p. 209) differentiates emotions employed as capital from other capital forms that are more durable or stagnant. The fact that emotional capital is so tightly linked to the interaction order means that it is more easily transferable between fields, since an ability for emotional attuning creates a sensitivity to interpret “what is going on” across fields, and thus also a potential to defy structural positions. However, the interactional dependency also makes emotional capital more volatile than other capital forms since it needs to be negotiated in every situation (Blumer, 1969).

To sum up, we propose that emotional capital should be understood as interdependent and defined as emotion-based knowledge, embedded within the other capital forms and relying on performance, interpretation, and conversion. With this notion, we do not limit emotional capital to gendered practices (although understandably women are taught to manage emotions in different ways than men) nor do we consider emotion as capital through use-value. Arguably, any capital needs an exchange value to even be considered as capital in the first place (c.f. Skeggs and Adkins, 2004; Wetherell, 2012). Next, we turn to previous research, tracing the changing conceptualization of this capital form.

### The changing conceptualization of emotional capital

The first strategic use of emotional capital can be traced back to Helga Nowotny who argues that emotional capital consists of love, care, affection and empathy; defined as “knowledge, contacts and relations as well as access to emotionally valued skills and assets” (Nowotny, 1981, p. 148). Nowotny views emotional capital as a female variant of social capital particularly emergent from and useful within the private sphere. Allatt (1993), building on Nowotny, similarly views emotional capital as more easily attainable for women than for men, arguing that emotional capital consists of special attention, care and concern which, according to Bourdieu, is built over time. Both these conceptualizations place emotional capital in the private sphere tightly connected to primary socialization. For Nowotny, emotional capital can be seen as an obstacle when moving from the private to the public sphere, as it only holds value in the first. It is important to note that Allatt does not restrict emotional capital as available only for women, but this early feminization of emotional capital has somewhat settled the realm in which emotional capital is studied.

In a study on mothers' involvement in their children's schooling, Reay (2000) stays within the private sphere, but shows how emotional capital can be utilized on the educational marketplace. By moving away from the focus on “feelings of care,” i.e., feelings that have normatively positive connotations, Reay shows that negatively connoted emotions, such as anxiety, actually can prompt children's educational efforts, albeit, sometimes at the cost of the wellbeing of both the mother and the child. Reay problematizes the outcome of utilizing emotional capital, but like others, limits the range of emotional expressivity to include only strong and visible emotions, omitting more subtle and less expressive emotions.

The restriction of emotions associated with the capital form, as we will see, has remained when the concept was transferred into other spheres, mainly education and nursing including men to a greater degree (Reay, 2000; Zembylas, 2007; Cottingham, 2016). This delineation seems to be caused by a combination of a theoretical association of emotion to the private sphere and an empirical focus on caring occupations. However, as we illustrate below, since there is nothing inherently caring about utilizing emotions as capital, including a wider range of emotions could deepen our understanding of how emotional capital works in interaction. Accordingly, Cottingham argues that emotional capital “is neither wholly gender-neutral nor exclusively feminine” (Cottingham, 2016, p. 451); it can be employed differently by men and women, but is not restricted to either sex. This articulation of the dependence of emotional capital on relational structures opens up for researching structural constraints beyond gender, possibly incorporating ethnicity (Wingfield, 2021) and class (Skeggs and Adkins, 2004). Another important qualification by Cottingham is that emotional capital should be analyzed as a resource, and thus does not necessarily have to be utilized to be considered a capital. It is the access to emotions as capital that matters, not whether it is made use of or not (Cottingham, 2016). Moreover, it raises the question of how this resource is acquired and whether it can be converted into other capital forms.

The expansion of empirical research to include work life indicates that although emotional capital is seen as primarily fostered in primary socialization it can also be acquired and cultivated in secondary socialization (Cottingham, 2016), making it more dynamic and susceptible to a wider range of change and adaption. The contextual adaption of emotional capital has been further developed by Heaney (2019) who argues that as a dynamic resource, emotional capital depends on the cultural and historical context within which it is employed. This means that emotional capital can decrease or increase when norms change. Placing emotional capital in a norm system actualizes its link and potential overlap with emotion management. Both Cottingham and Heaney argue that emotional capital is different from emotion management since capital, in Bourdieu's vocabulary, is more of a “feel for the game,” an order without outspoken rules (Bourdieu, 1990; c.f. Heaney, 2019) or rational calculation (Cottingham, 2016). Although, emotion management in Hochschild's original version emphasized the deliberate use of emotions in capitalist systems, her concept of labor has a Marxist origin, linking the experience and expression of emotion for commercial goals to transmutation; labored emotions, with time, form habitual patterns without need for outspoken rules. Later research has widened emotion management to also include interpersonal adaption within organizations (Fineman, 2003) and in private life (Illouz, 2007), where habitual patterns are dominant. In this way, emotion management can be deliberate or habituated and thus link both to outspoken and tacit rules.

Instead, we argue that the important difference between emotional capital and emotion management is that emotion management often reproduces subordination and status quo (Hochschild, 2009). As Lively (2002) argues in a study on paralegals, female paralegals need to harbor emotions of both clients and lawyers, and their high demands for emotion management reproduces their subordinate position. Their emotional competence might make them socially valued, but it has no exchange value into other capital forms. Similarly, in Skeggs' study on working class mothers, she found that their emotion management only produced “use-value” (Skeggs and Adkins, 2004), denoting emotion used as a response to social conditions. In other words, this “could not be described as building emotional capital in any conventional sense because there is no “trading up” to create [the] surplus value” (Wetherell, 2012, p. 114) which is essential to be considered capital. As hinted at by these examples, the capacity to turn emotion management into capital is stratified. While a white man's display of anger can signal potent leadership (Pierce, 1995), a similar expression by a woman or black man is more likely to signal an unbalanced personality (Sheilds, 2002) or propensity for violence (Wingfield, 2021). However, the interaction order as a stand-alone construct implies that the importance of these structural constraints may vary depending on the situation.

When emotional displays and emotion management can be employed as capital, it should be able to create surplus or symbolic value across fields to a larger extent than cultural capital that demands knowledge related to a specific field. Emotional capital is in this sense both stable and volatile. It takes time to accumulate since it relies on a “feel for the game,” but ability to employ emotions as capital by for example caring or displaying confidence and ease settles in the body, and as accumulated labor can transfers across fields. The accumulated emotional capital of a judge on the bench can award status and permission (elite distinction) to display a wider range of emotions in the supermarket on the way home from work or at the parental meeting at her children's school. It is volatile in that it depends on the interaction order making the feel for the game by necessity a situational accomplishment. As we will show, participants deviating from expected emotion norms can, at least momentarily, disturb or overthrow hierarchal positions by not acknowledging the symbolic value of the displayed emotion since situational interpretations miss-match.

To recapitulate, we agree with Cottingham's definition of emotional capital as including emotion management, often in a less conscious, habituated form, but this “feel for the game” (Bourdieu, 1990, p. 63) needs to be a resource that can be utilized to reproduce or advance one's position within a social space in order to be defined as capital. The benefit of articulating and delineating the relation between emotion management and emotional capital and in particular their link to a norm system is that the dynamic and to some extent volatile nature of this capital form comes to the fore. Since norms and relational structures can play out differently in different fields, there is a need to look more closely at how interactional and situational orders influence the ability and power to utilize emotional capital.

To sum up, we can see an evolution of the concept of emotional capital from being linked to social capital and primary socialization, to being connected to cultural capital, with an ability for continuous transformation in secondary socialization. The utilization of emotional capital so far has been largely restricted to “feelings of care” as mirrored in the professions studied. We argue that this narrow application of emotional capital disregards some interesting angles that can be elicited from the well-rounded definition provided by Cottingham. As a result, the following will deal explicitly with how emotional capital can be understood as including more than just “feelings of care” and how it intermingles with power in interaction. First, let us introduce the material employed in the analysis.

### Methods and data

To elaborate on how emotional capital is used in practice we draw on material from a larger ongoing international project, JUSTEMOTIONS, investigating objective legal decision-making through a multimethod qualitative approach of observations, interviews and shadowing in Sweden, Italy, USA and Scotland. The project has been approved by ethics committees in all four countries and by the European Research Council.

The analysis in this article builds on data from two of the participating countries, Scotland and the United States. These countries were chosen for their high prevalence of informal negotiations between legal professionals, such as plea bargains, which makes for a rich variation of both formal and informal social interactions between multiple actors. Data consists of observations of proceedings and informal interactions in court combined with interviews with 51 participants, 26 judges and 25 prosecutors. The participants, 50% women, were between 24 and 74 years old (with a mean of 48 years), with experience ranging from under 1 year to over 30 years^1^. During the court observations, the first author shadowed participants throughout their workday including both formal and informal proceedings and preparation. The continuous change between active and non-active participation during shadowing demanded tact and adaption by the researcher, but also paved the way for inter-personal trust and openness in both informal conversations and interviews. The semi-structured interviews, between 50 to 120 mins long, took place in connection with the shadowing and observations allowing for questions about recently observed interactions.

In the analysis, we first identified moments of interaction with a specific focus on social bonding and conflict since these put the emotional dynamic to the fore and demand (more or less reflected) strategy. Secondly, focus was placed on identifying the different emotional responses at play and how they were expressed and managed. Third, we explored the development and outcome of the interactions from a power and status perspective in relation to the participating actors. The data presented here, from observations, shadowing and interviews, was collected from fall 2019 through fall 2020. In the selected quotes, we present the participants with professional role, fictitious name, age, self-reported ethnicity, court level, and country. Since heterogeneous career paths and a propensity to switch between professional roles in court make time spent in the current role a precarious indicator of work experience, we use age as a coarse but more reliable proxy.

### Emotional capital in courtroom interactions

In the following, we demonstrate how emotional capital is actualized, employed, and converted in interaction. We argue that a wider range of emotion is available to capitalize on, and that the inherent volatility of this capital form indicates its dependency on the interaction order, which becomes particularly prevalent in complex interactions where emotions, status positions and interactional challenges intermingle.

#### Expanding the range of emotions in emotional capital—Ease and irritation

Previous research has shown that feelings of care often constitute emotional capital. We have found that two other emotions dominate our material, namely ease and irritation. This is unsurprising, since both of these emotions are traditionally connoted to the elite sphere but, as we shall see, they can also generate capital. Ease, because it can be considered a rational emotion (James, 1879); irritation, as it is linked to status (Kemper, 2006).

In the following quote, we meet Logan, a High Court judge in Scotland, who reflects on the mismatch between his own feelings of ease in the court setting and the contrasting discomfort felt by the majority appearing in court.

I feel at ease in a court, but for nearly everyone who comes into the court […] they'd probably not rather be there. […] I view it as a very, very important part of our job to be sensitive and responsive to the difficulties that ordinary people face coming in to this very intimidating environment. So I'm looking out for that all the time. I try hard. Particularly with witnesses and members of the jury to put them at ease […]. A judge doesn't have to spend very long to do that. Just a smile of welcome, minor courtesies ‘would you like to sit down, you don't have to stand' and just telling people what's going to happen and giving them an understanding of what's about to happen (Judge Logan, 50+, Caucasian, Interview, High Court, Scotland).

The courtroom is a routine space for Logan who spends much of his professional time there. “Ease is a feeling …of the sufficiency of the present moment, of its absoluteness—this absence of all need to explain it, account for it or justify it…” (James, 1879, p. 317). Ease is considered a central emotion for signifying elite distinction (Bourdieu, 2018). Logan acknowledges the fact that most people visiting the court sees it as a “very intimidating environment” and Logan's feeling of ease becomes (accumulated) capital in itself, but he also employs his own ease as an empathic capacity, a contrast to notice and understand any discomfort or fear in visitors. He tries to be sensitive to others' experiences (“I'm looking out for that all the time”). To feel at ease in court requires a cultural capital that few people visiting the courts have acquired, and Logan takes pride in employing his own ease and knowhow to tone down intimidation and facilitate the court interaction. As we can see, Logan's own ease and empathic attuning with the people visiting his court can be translated into emotional capital in that it reinforces his status. In this way, Logan can convert his emotional capital into symbolic capital, because he is seen as an impartial representative of the state (Roach Anleu and Mack, 2021). In his position as a judge, Logan represents and serves society and, in the longer run, his impartial, and empathic, display serves to reproduce public trust in the legal system and the rule of law, in effect linking repeated micro-interactions to macro effects.

In the following, we will show examples where instead feelings of irritation upholds the interaction order. First, we meet Judge Ewan from a rural Sheriffs Court in Scotland. The excerpt shows an interaction between Judge Ewan and the prosecutor, who is arguing for the use of a video link in an upcoming testimony. The prosecutor uses a legal principle to justify his request, but is interrupted by Ewan.

“I don't think I am understanding what you are saying. You're the prosecutor representing the crown in this case so I think I am allowed to ask. As you can see I have the principle in front of me and I thought you would have done the same.” Ewan seems annoyed now. He is picking at the papers harder, and speaks with a direct and stern voice as he says “the crown's obsession with electronic devices seems to make it worse rather than better.” […]. The prosecutor starts, but is interrupted again, Ewan is now leaned back and says [he] should have things cleared up before he asks the court for something (Sheriff Ewan, 60+, white, Observation, Sheriffs Court, Scotland).

As we can see, there is a lack of understanding between the parties and a conflict of interpretation (Bourdieu, 1986a), which results in the ensuing exchange. Judge Ewan immediately defends his question by emphasizing the roles held in court (“You're the prosecutor […] so I think I am allowed to ask”). In this way, we learn that not only do the actors hold different roles in this interaction but also, questions should come from the judge, emphasizing the status difference between them. Irritation is driving Ewan's interruptions (“Ewan seems annoyed now. He is picking at the papers harder”) and he finishes by emphasizing the proper way to approach the court (“have things cleared up before [you] ask the court”). Irritation and anger are common emotions for judges' faced with poor preparation (Maroney, 2012), often employed as emotional capital. By his explicit use of irritation, Ewan is able to call attention to his own professional expectations as well as those of the court. Previous research has shown that prosecutors and lawyers are sensitive to expressions of irritation from the judge and adapt their behavior accordingly not to risk unfavorable judgments (Bergman Blix and Wettergren, 2018a)—irritation and ease thus become capital in a status setting as it changes the course of events and the behavior of other actors (Kemper, 2006).

#### Interactional challenges

When Judge Ewan or Logan used their emotional capital, they did not receive any pushback from the other actors; but such compliance is not always achieved. In the next example we meet Nathania, a seasoned judge in a US court, dealing with orders of protections. In these hearings, laypeople often represent themselves, which can demand more participation from the judge. In the extract below, Judge Nathania is trying to assess a situation by asking questions to a mother, but the father keeps interrupting.

The child's father starts talking but Nathania interrupts him “I don't know why you are interjecting, maybe because I looked at you, I don't know.” […]. Her voice is genuine, but unsure. The mother continues, explaining that she has not heard from her child's father in 7 years. The child's father interrupts again and Nathania says “would you be quiet until I'm ready to talk with you?” Her voice is stern now and she immediately turns her attention back to the mother. […] Nathania starts “okay what…” but is interrupted again. She now turns her whole body toward the side of the courtroom where the father is standing and says “ah, ah I'm talking, you're not gonna out-talk me in here, you can't control me!” [high pitch]. He responds in a lower voice “I'm not trying to control you.” […]. He is getting emotional, his voice is now louder and wavering, “I miss my child” […]. Nathania continues, “you're still trying to out talk me.” She looks straight at me [the researcher] and scoffs. […]. Nathania then just says “I'm gonna put this order of protection into effect. You're aggressive…” (Judge Nathania, 50+, black, Observation, First Instance Court, United States)

The father's inability or resistance to abide by the interactional rules threatens Judge Nathania's status and her ability to run a smooth hearing. When challenged, Judge Nathania employs different emotions to restore the interaction order, moving from the use of empathy (“maybe because I looked at you?”) to the more confrontational emotion of irritation. Although her emotional capital relies on her stable cultural capital, her social position is not enough to manage the situation. The disagreement on how to interpret the situation exposes the epistemic gap between a lay understanding and a legal understanding of “what is going on here” (Goffman, 1986, p. 8). That Nathania fails to mend the gap by employing emotional capital could be explained by a mismatch in their respective situational definitions. Nathania commits to presiding in court and securing procedural rules, while the father displays moral commitment to his social performance as a father. As Rawls argues, “the display of moral behavior by members of one group may well look like deviant behavior by members of the other” (Rawls, 2000, p. 247). The colliding interaction orders momentarily disrupts the hearing despite Nathania's high status position and situational knowledge. Although Nathania eventually restores order by falling back on a power display (deciding on a protection order before having heard both sides), her interactional failure apparently makes her insecure and exposes her vulnerability (“you can't control me!”). Using power momentarily devalues her status (Abbott, 1981) and puts her legitimacy at risk. For the judge's power to be found legitimate, she depends not only on cultural capital in the form of legal Bildung (Bourdieu, 1986b) but also on emotional capital to gain enough situational status to run a correct procedure and derive valid material for legal interpretation.

Next, we enter Judge Blake's court. Blake is straightforward in his role as a judge; he has over 30+ years' experience and runs his court with equal parts of humor and dressing-downs. The following shows the latter:

[In an adjacent interview room] A prosecutor comes in and says the judge is on the bench, they all rush back to the courtroom. As we walk in Judge Blake says “Now, Generals, it's imperative that when I'm on the bench conducting business for the state that you be in here.” His voice is stern and he raises a finger as we take a seat.

Judge Blake expects legal professionals (and laypeople) in his court to be alert to him and he frequently reprimands prosecutors and attorneys using both his voice and body (employing the military reference “Generals” with a stern voice and raised finger), in effect reproducing or strengthening his power in court. However, at another occasion, when faced with a different interruption, his demeanor changes. The case concerns an accusation of intimate partner violence involving a man and a woman. The woman is the defendant in the case currently on the docket, but following her own arrest, she has taken out a cross-warrant on the man who accused her of domestic violence. When we enter the situation, the defense attorney is shouting at the judge in an angry voice accusing the prosecutor of being racist for dismissing the case involving the female defendant.

The judge stops and answers the defense lawyer with a soft voice. He says that he does “not think that the prosecutor is acting on racist grounds” but that he “absolutely wants to hear what the defense lawyer has got to say.” The judge keeps eye contact with the defense lawyer, only looking away when speaking of the prosecutor […]. The defense attorney reminds the court that the alleged victim [the woman] was arrested first, and it was not until after her arrest that she accused her partner. With this in mind, the defense attorney argues, still in an angry tone, that the case cannot be resolved as it is. The judge leans back and asks if the male defendant is present today and, if so, he should be brought in, so that the court can hear both sides of the story. The defense lawyer seems to view this as an appropriate solution as she leaves to bring her client (Judge Blake, 60+, white, Observation, First Instance Court, United States).

The hearing takes place during the Black Lives Matter demonstrations in 2020 placing racial injustice top of mind in cases relating to this issue on different court dockets across the United States (Hurley, 2020). In the present case, the female defense lawyer is black representing a black man, while the prosecutor, the judge and the discharged female defendant are all white. When the court faces the accusation of racism, Judge Blake's demeanor changes instantly (“with a soft voice”). Although Blake disagrees with the defense lawyer (“the prosecutor is [not] acting on racist grounds”), he is willing to change the order of business to address her concerns (“[the defendant] should be brought in”). The changes, however, are not only organizational but also refer to demeanor. One can assume that Judge Blake changes his demeanor since the defense lawyer herself holds the cultural capital needed to argue that discrimination, based on race, has taken place. It is through her cultural capital that her anger can be used as emotional capital. In other words, the irritation shown by Blake in the first quote would not constitute capital in this situation, when the interaction order is challenged. In the face of changing norms (Heaney, 2019), the defense attorney can convert her emotional capital into symbolic capital. Apparently, Blake's performance here can also be viewed as grounded in emotional capital, as he restores the interaction order by adapting his emotional display to manage the defense attorney's anger.

Although our sampling and data do not allow for systematic analysis of gender or racial differences, we can note that Nathania, a female black judge, failed in using an empathic perspective and soft voice to cool down a situation, while the same strategy used by the white male judge restored the interaction order as well as his status.

Another way to handle potential conflicts or just ensure a smooth procedure is to use humor (Scarduzio, 2011). When Nathania, the judge above who failed to employ emotional capital, got complains from a defense attorney for taking a short break, she expressed irritation when leaving the room, but when she re-entered she turned to the defense lawyer on her way up on the bench and asks with a sassy voice: “Was that quick enough for you Ms X?” The defense lawyer replied: “super quick!” to which Nathania answers with a smile, now more sarcastically, “I always try to please” (Nathania, 50+, black, observation, First Instance Court, United States). Using humor to restore a tense or bored atmosphere can induce a sense of solidarity and equality (c.f. Bergman Blix and Wettergren, 2018b). Importantly, when fun is deemed appropriate, the funny person gains in status (Robinson and Smith-Lovin, 2001). Nathania's management of her irritation into a playful display worked as emotional capital in that it restored her status position and gave her leeway to continue presiding in an efficient way.

#### Emotional capital as a collective achievement

As we saw in the last example, emotional capital can depend on collaboration to be successfully employed. Social position and interactional challenges intermingle with the need to cooperate to facilitate the process. Next, we meet Prosecutor Rebecca, a senior prosecutor in a US criminal court handling a case of domestic abuse, rape and kidnapping. We enter the courtroom during the cross-examination by the defense at the preliminary hearing. There is an ongoing discussion involving the prosecutor, the judge and the defense lawyer on whether or not to bring up a previous charge concerning the same defendant and the alleged victim in which the victim, during trial, denied having been abused, eventually leading to the case being acquitted. The defense have tried to bring this up several times to undermine her current testimony, whilst trying to gather additional evidence for the defendants innocence by asking access to the witness' text messages, but Rebecca continues to object to his line of questioning and explaining her reasons below.

Rebecca stands and says, “I don't want her to relive that, since we are in court on the current charges” [irritation]. The judge turns to the defense lawyer and says, “The court is *very well aware* of how domestic violence works [sarcastically]” he puts his hand up as the defense lawyer starts talking, and says, “Let's stay on point.” The defense lawyer says, “When you claimed…” The judge sighs and Rebecca scoffs whilst looking at each other. The witness speaks up [with emphasis], “I lied because I was *scared!*” […]. The defense lawyer pauses as the witness starts crying, her hands are shaking. After a few seconds, Rebecca stands and asks the defense lawyer to finish so that the witness can get down from the stand. The defense lawyer quickly turns to Rebecca, and says, “I'm just giving her a minute” [impatient]. The judge turns to the witness and asks if she is okay to continue for a few minutes. When she says yes, the defense lawyer asks about her cell phone. Rebecca sighs, still standing, and mutters “Oh God [disbelief]” The defense lawyer then turns to the prosecutor and tells her “the state needs to provide the text messages from the cell phone,” in a stern voice. Rebecca stands, chuckles and says, “Just to clarify, we are underway to try to retrieve the messages but I cannot be sure yet what is forensically possible.” The judge again, “they are retrievable at least from my experience [pause] and I don't know a lot about technology” [laughter] (Prosecutor Rebecca, 50+, Caucasian, Observation, First Instance Court, United States).

The interaction above is interesting for several reasons. The conflict stems from the fact that the defense might benefit from bringing up the previous (acquitted) offense, while the prosecution wants to keep such questioning at bay not to endanger the credibility of the witness. If we focus on how these challenges are handled emotionally and strategically by the different parties, we note that Prosecutor Rebecca reinforces her argument emotionally using irritation (recurrently standing up and interrupting the defense lawyers) and gains support from the judge (“the court is *very well aware* of how domestic violence works”). The emotional back up from the judge allows Rebecca to continue interrupting. In the first part of the interaction, the judge and prosecutor collectively employ emotional capital, using empathy, sarcasm and frustration (“The judge sighs and Rebecca scoffs”), leaning on experience and ultimately status, and are able to move the process forward in their preferred way. The defense lawyer is forced to adjust to their demands, but keeps trying to keep up his status, first by framing his approach as considerate and then by turning on the prosecutor with a formal request (about the cell phone). Here, he gets the judge's attention and the table turns in his favor. The judge's foundational requirement to act impartially implies that he continually needs to shift the direction of his emotional capital. His toning down of his expertise on technical issues and accompanied laughter smooths the transition and bolsters the emotional let-down for the prosecutor.

As we can see, the legal actors rely on each other's cooperation to employ emotional capital, underscoring the importance of the interactional order for understanding this capital form. By emotionally tuning in and siding with first the victim and the prosecutor, and then the defense, the judge manages to maintain and, in the longer run, build his status as a fair and impartial judge. This demonstrates the importance of empathy for emotional capitalization also for high status actors, especially in professions requiring impartiality. The prosecutor and defense both try to win the judge over, but also need to stand their ground when they fail, maintaining and building their status by signaling partial objectivity (prosecutor) (Bergman Blix and Wettergren, 2018a) and loyalty to their client (defense) (Flower, 2018). Rebecca's ability to know when to push and when to pull back signals professional experience and ease, but the constant shift between overlapping and conflicting goals for the participating professionals also highlight the interactional volatility of emotional capital for high status actors with secure individual as well as institutional resources. In contrast to the potential mismatches in defining the situation between lay- and professional actors, this interprofessional interaction counts on a shared order, but still involves conflicting interpretations for strategic reasons. Successful employment of emotional capital in this situation depend on interpersonal emotional attuning and support.

## Conclusion

In this article, we have used the example of high status legal professionals to illustrate on the one hand the interactional volatility of emotional capital, and on the other, a wider range of emotions that can be employed than has been found by previous research. We demonstrate that emotional capital is employed through emotions such as ease, irritation and reluctance as well as empathy. These emotions are readily available and “fitting” to legal professionals as a result of professional socialization (Cottingham, 2016). When risking their status, the legal professionals on the other hand, rely on a “feel for the game” (Bourdieu, 1990, p. 63) building on emotional sensitivity to tune into the situation, humor, or aggression, since failure to employ one's emotional capital can lead to loss of legitimacy. In other words, emotional capital can reinstate actors' social position when the interaction or the participants' status is challenged.

Although “feelings of care” are present also in these professions, they are often accompanied by a variety of emotions, making use of emotional capital a more complex balancing act than previously asserted. High status actors cannot only fall back on their capital wealth but need to employ strategic emotion management to uphold or restore their status and ability to employ their emotions as capital. It is also interesting to note that in these high status governmental positions, the conversion of emotional capital into symbolic capital implies securing and reproducing societal trust. The performance of an objective prosecutor or an impartial judge demands utilizing emotional capital that, with time, becomes a recognized positional competence. As such, successful employment of emotional capital does not only reinforce the legal professionals' individual status but also the legitimation of the legal institution.

Emotional capital relies on other forms of capital and a retreat to more robust capital forms such as cultural capital can be used to re-evaluate the interaction and reinstitute the status structures present in the specific field, for example by falling back on legal terminology and procedure. However, when this happens, actors often have to articulate their hierarchical positions actively using their power at the expense of their situational status (Abbott, 1981). Notably, by employing emotional capital skillfully, the interactional space can become more available to any actor regardless of other capital resources.

The increasing need to account for emotional capital in high status bureaucratic professions aligns with larger societal changes where bureaucracies are becoming more service centered (Du Gay, 2008) together with a shift in elite distinction toward ordinariness and informality (Friedman and Reeves, 2020). Although this invites an epistemic bridge between professionals and lay people where empathic perspective taking becomes a more generic quality, it also opens up for risks for high status actors of being challenged to a greater degree. Professional adherence to strict rituals such as a court procedure where the stakes are high for laypersons increases the risk for collision between professional expectations and personal needs and thus, mismatching interaction orders.

It is interesting to note that, in contrast to early studies that saw emotional capital as a resource for women lacking other capital resources; our examples from an elite profession demonstrate a need to employ emotion as capital despite overall capital wealth and high status. Although not systematically analyzed, we identified little pushback for male and white professionals when employing emotions as capital. This highlights the need for further studies to explore the constraining impact of gender and race operating within the interaction order. All legal professionals needed to attune emotionally to the situation and manage potential interaction order mismatches to uphold their status in court, but they may be differently restrained in relation to which emotions they can capitalize on.

## Data availability statement

The datasets presented in this article are not readily available because part of the data is confidential (observation of preliminary investigations and deliberations). Questions about the dataset should be directed to stina.bergmanblix@uu.se.

## Ethics statement

The studies involving human participants were reviewed and approved by Uppsala Regional Ethics Committee (2017/284), Vanderbilt Institutional Review Board (191725), and University of Strathclyde Ethics Committee (UEC2019/14). Ethical approval by the European Research Council. Verbal or written consent has been acquired from all participants.

## Author contributions

CN: conception and acquisition of data. SBB: obtaining funding, conception, and design of research. SBB and CN: article design, analysis, interpretation of data, drafting, and revising manuscript. All authors contributed to the article and approved the submitted version.

## Footnotes

^1^Fifteen female and nine male prosecutors; 11 female and 13 male judges. The majority of the participants identified as Caucasian/white, six identified as black/African American and three as Hispanic/Latinx.
